# Extended notions of sign consistency to relate experimental data to signaling and regulatory network topologies

**DOI:** 10.1186/s12859-015-0733-7

**Published:** 2015-10-28

**Authors:** Sven Thiele, Luca Cerone, Julio Saez-Rodriguez, Anne Siegel, Carito Guziołowski, Steffen Klamt

**Affiliations:** 1 Max Planck Institute for Dynamics of Complex Technical Systems, Magdeburg, 39106 Germany; 2 European Molecular Biology Laboratory, European Bioinformatics Institute, Hinxton CB101SD, UK; 3 CNRS, UMR 6074 IRISA, Campus de Beaulieu, Rennes, 35042 France; 4 INRIA, Dyliss project, Campus de Beaulieu, Rennes, 35042 France; 5 École Centrale de Nantes, IRCCyN UMR 6597, 1 rue de la Noë, Nantes, 44321 France

**Keywords:** E. coli, Gene regulation, Interaction graphs, Sign consistency, Uncertainty, Logic modeling, Answer Set Programming (ASP)

## Abstract

**Background:**

A rapidly growing amount of knowledge about signaling and gene regulatory networks is available in databases such as KEGG, Reactome, or RegulonDB. There is an increasing need to relate this knowledge to high-throughput data in order to (in)validate network topologies or to decide which interactions are present or inactive in a given cell type under a particular environmental condition. Interaction graphs provide a suitable representation of cellular networks with information flows and methods based on sign consistency approaches have been shown to be valuable tools to (i) predict qualitative responses, (ii) to test the consistency of network topologies and experimental data, and (iii) to apply repair operations to the network model suggesting missing or wrong interactions.

**Results:**

We present a framework to unify different notions of sign consistency and propose a refined method for data discretization that considers uncertainties in experimental profiles. We furthermore introduce a new constraint to filter undesired model behaviors induced by positive feedback loops. Finally, we generalize the way predictions can be made by the sign consistency approach. In particular, we distinguish strong predictions (e.g. increase of a node level) and weak predictions (e.g., node level increases or remains unchanged) enlarging the overall predictive power of the approach. We then demonstrate the applicability of our framework by confronting a large-scale gene regulatory network model of *Escherichia coli* with high-throughput transcriptomic measurements.

**Conclusion:**

Overall, our work enhances the flexibility and power of the sign consistency approach for the prediction of the behavior of signaling and gene regulatory networks and, more generally, for the validation and inference of these networks

**Electronic supplementary material:**

The online version of this article (doi:10.1186/s12859-015-0733-7) contains supplementary material, which is available to authorized users.

## Background

The advancements of measurement technologies and high-throughput methods in molecular biology have led to a tremendous increase in the availability of factual biological knowledge as well as of data capturing the response of biological systems to experimental conditions. Knowledge about metabolic, signaling, and gene regulatory interactions and networks is available in databases such as KEGG, Regulon DB, PID, or Reactome which can be used as a starting point to build causal models of bio-molecular networks [1]. Specifically, signaling and gene regulatory networks carrying signal and information flows can be represented as interaction (or influence) graphs [2–6], Bayesian networks [7], some form of logic (including Boolean or constrained fuzzy logic) modeling [4, 8, 9], or ordinary differential equations [10–12]. However, there is an increasing need to relate large-scale network models to high-throughput data in order to (in)validate network topologies or to decide which regulatory or signaling interactions are present in a particular biological system, cell type, environmental condition etc.

Significant work has been published on this subject, attempting to detect inconsistencies among measured high-throughput data and signaling and regulatory networks and to subsequently identify missing or inactive interactions such that the optimized network structure maximizes consistency with experimental data [2, 4, 13–18]. Some of these approaches use signed directed graphs, also called interaction or influence graphs (IG), as underlying model where edges indicate either positive or negative effect of one node upon another. Although these models are qualitative and simple, they have frequently been used to study signal flows in a wide range of biological systems. Moreover, the fact that every Boolean and every ODE model has an underlying interaction graph renders their analysis directly relevant for other modeling formalisms and it has been shown that some important global properties of Boolean or ODE models are determined by the structure of their associated IG [6, 19, 20]. IG have also been used for qualitative reasoning, to describe physical systems where a detailed quantitative description is unavailable [21]. In fact, this has been one motivation for using IG in the context of biological systems [20] where knowledge and data are usually uncertain.

One important class of methods relating IG with experimental data is based on the notion of *sign consistency*. The key idea here is to represent the potential network behaviors resulting from steady-state shift experiments (such as upregulation or downregulation of node activation levels after network perturbations) by certain kinds of discrete constraints. A first approach based on sign consistency was introduced in [2]. There, experimentally measured changes in node activities were represented by two labels (increase, decrease) on the IG nodes. Constraints relating nodes labels and IG are introduced to model the propagation of regulatory effects. Later, in [3, 22], Answer Set Programming (ASP) [23] was used to find admissible node labelings adhering to the posed constraints, and optimal repairs to restore sign-consistency were proposed. A related formalism was presented in [17]. Major differences to previous studies were (i) consideration of three node labels (increase, decrease, 0-change), (ii) the representation of the constraints as an integer linear programming (ILP) problem, and (iii) the introduction of new repair operations minimizing inconsistencies between the IG structure and the experiments.

The goal of this study is fourfold. First, we aim at unifying existing approaches into a general framework. We show that different notions of sign consistency mainly differ in the way zero changes are modeled. Then, we propose a refined method for data discretization allowing one to express uncertainties during the discretization step. In addition, we introduce a new constraint to filter undesired self-fulfilled explanations which result from positive feedback loops. Finally, we introduce an extended prediction method that allows not only strong (e.g., "increase") but also weak predictions (e.g., "increase or 0-change"), enlarging the predictive power of the approach. We applied the extended framework to a realistic case study where we analyze high-throughput transcriptomic measurements of *Escherichia coli* in the context of a large-scale gene regulatory network model obtained from RegulonDB. Taken together, we demonstrate that these extensions increase the applicability and flexibility of the approach significantly.

## Methods

### Definitions

**Fig. 1:**
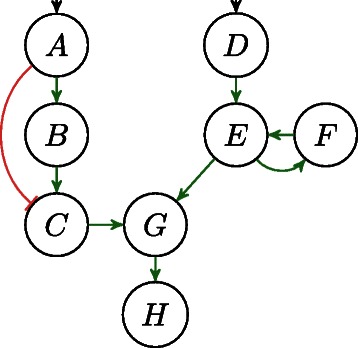
Interaction graph with a positive feedback loop between *E* and *F*

An *influence or interaction graph* (IG) is a signed directed graph (*V*,*E*,*σ*), where *V* is a set of nodes, *E* a set of edges, and *σ*:*E*→{+,−} a labeling of the edges. Every node in *V* represents a species in the modeled system and an edge *j* → *i* means that the change of *j* in time influences the level of *i*. Every edge *j* → *i* of an IG can be labeled with a sign, either + or −, denoted by *σ*(*j*,*i*), where + (−) indicates that *j* tends to increase (decrease) *i*. An example IG is given in Fig. 1.

In this framework, we confront the IG with *experimental profiles*. In our approach, the experimental profiles are supposed to come from steady-state shift experiments where, initially, the system is at steady-state, then externally perturbed in certain nodes, and settles eventually into another steady-state. For some species *S*⊆*V* (genes, proteins, or metabolites) concentrations are measured in the initial and final state. The raw data is given by a real value *o* *b* *s*(*s*) for every measured species *s*∈*S* specifying the difference of the node states at the beginning and in the new steady state. As defined below, we determine for these nodes whether the concentration has increased, decreased or not significantly changed.

### Data discretization

**Fig. 2:**
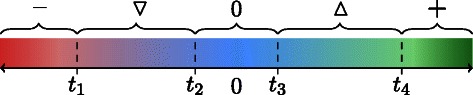
Discretization of observed changes into sign constraints

We propose a refined method to discretize the measurements using four (condition-dependent) thresholds *t*_1_≤*t*_2_<0<*t*_3_≤*t*_4_, allowing one to consider uncertainties in the discretization process. As illustrated in Fig. 2, these thresholds define a mapping $\mu : S \rightarrow \{{-}, \triangledown, 0, \vartriangle, + \}$ as follows:

$$\mu(s)=\left \{ \begin{array}{lll} {-} & |\quad & obs(s) \leq t_{1}, \\ \triangledown & |\quad t_{1} < & obs(s) \leq t_{2},\\ 0 & |\quad t_{2} < & obs(s) < t_{3}, \\ \vartriangle & |\quad t_{3} \leq & obs(s) < t_{4},\\ + & |\quad t_{4} \leq & obs(s). \end{array} \right. $$

We consider measurements which are smaller than *t*_1_, bigger than *t*_4_, and between *t*_2_ and *t*_3_ as certain (decrease -, increase +, no-change 0) while measurements that are between *t*_1_ and *t*_2_ (resp. *t*_3_ and *t*_4_) are uncertain (uncertain-decrease $\triangledown $, uncertain-increase $\vartriangle $) and not exactly classifiable. With that, an experimental profile (*S*,*I*,*μ*) is defined by the set of measured species *S*, the set of *input nodes* *I*⊆*S* (the experimentally perturbed species) whose changes are trivially explained, and the mapping *μ* as defined above.

### Local consistency rules

Given an IG (*V*,*E*,*σ*) and an experimental profile (*S*,*I*,*μ*) one can describe the rules that relate both. For this purpose we look for total labelings *μ* ^*t*^:*V*→{−,0,+} that satisfy the local constraints defined below. It is important to notice that *μ* ^*t*^ will define a *total* labeling using the *three* labels {−,0,+} whereas *μ* defines a *partial* labeling (only measured nodes are labeled) based on the *five* labels $\{ {-}, \triangledown, 0, \vartriangle, {+} \}$ representing the discretized measurements.

With the first constraint, we look for total labelings *μ* ^*t*^ that satisfy the observed measurements captured in the partial node labeling given by *μ*:

#### **Constraint1** (satisfy observations).

Let (*V*,*E*,*σ*) be an IG, (*S*,*I*,*μ*) an experimental profile, *μ* ^*t*^:*V*→{+,−,0} be a total labeling, and let *i*∈*V* be a node with *μ* ^*t*^(*i*)∈{+,0,−}.

Then *μ* ^*t*^ satisfies Constraint 1 for node *i* iff *i*∉*S*, or *μ* ^*t*^(*i*)=+ and $\mu (i)\in \{ {+}, \vartriangle \}$, or *μ* ^*t*^(*i*)=0 and $\mu (i)\in \{ \vartriangle, 0, \triangledown \}$, or *μ* ^*t*^(*i*)=− and $\mu (i) \in \{ \triangledown, {-} \}$.

Note, uncertain measurements restrict the labeling of a node to two out of the three values {+,−,0}, while measurements with high certainty fix a node’s label to exactly one value.

Next we demand for every non-input node *i*, that its change *μ* ^*t*^(*i*) ought to be explained by the total influence of its predecessors in the IG. The *influence* of *j* on *i* is given by the product *μ* ^*t*^(*j*)*σ*(*j*,*i*)∈{+,−,0}.

#### **Constraint2** (change must be justified by a change in a predecessor).

Let (*V*,*E*,*σ*) be an IG, (*S*,*I*,*μ*) an experimental profile, *μ* ^*t*^:*V*→{+,−,0} be a total labeling, and let *i*∈*V*∖*I* be a non-input node with *μ* ^*t*^(*i*)∈{+,−}.

Then *μ* ^*t*^ satisfies Constraint 2 for node *i* if there is some edge *j* → *i* in *E* such that *μ* ^*t*^(*i*)=*μ* ^*t*^(*j*)*σ*(*j*,*i*).

Constraint 2 is consistent with the propagation rule used in [2, 3] which demands that increases and decreases must be explained by predecessor nodes while 0-changes are unconstrained, that is 0-changes can always occur irrespective of the state of the predecessor nodes (note that 0-changes were not considered in [2, 3]). One argument for this reasoning is that it is often impossible to estimate the strength of the influences and the thresholds at which a downstream effect occurs are unknown. Hence, we cannot guarantee that an influence really has an effect and therefore allow 0-change. On the other hand, the constraint still enforces explanations for observed changes in node activation levels; each change must be explainable by an influence (with proper sign) of at least one predecessor.

Melas et al. [17] suggested also to demand proper explanations for 0-changes using the following constraint:

#### **Constraint3** (0-change must be justified).

Let (*V*,*E*,*σ*) be an IG, (*S*,*I*,*μ*) an experimental profile, *μ* ^*t*^:*V*→{+,−,0} be a total labeling, and let *i*∈*V*∖*I* be a non-input node with *μ* ^*t*^(*i*)=0. Then *μ* ^*t*^ satisfies Constraint 3 for node *i* if there is either no edge *j* → *i* in *E* such that *μ* ^*t*^(*j*)*σ*(*j*,*i*)∈{+,−} or there exist at least two edges *j*_1_ → *i* and *j*_2_ → *i* in *E* such that *μ* ^*t*^(*j*_1_)*σ*(*j*_1_,*i*)+*μ* ^*t*^(*j*_2_)*σ*(*j*_2_,*i*)=0

Constraint 3 restricts the occurrence of 0-changes. A node is only allowed to show 0-change if it receives either no influence or contradictory influences. This constraint thus assumes that each influence has indeed an effect and only contradictory influences can cancel each other out.

**Fig. 3:**
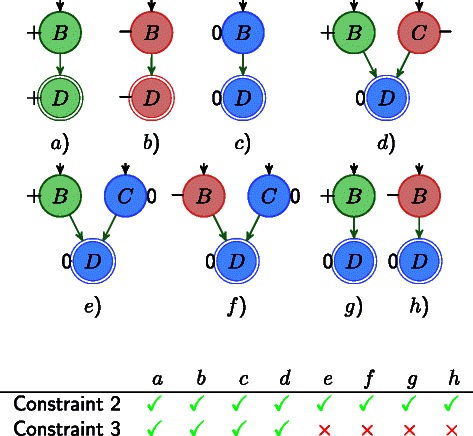
IGs with different labelings where green stands for increase, red for decrease, and blue for 0-change. All labelings satisfy the basis Constraint 2 for node *D*, but only the labelings *a*-*d* satisfy also Constraint 3. Examples with uncertain measurements are shown in the Additional file 1

In Fig. 3, we illustrate IGs with different labelings where green stands for increase, red for decrease and blue for 0-change. Notice, that Constraint 2 intentionally allows situations like in labeling *g* and *h*, where *D* is labeled as 0-change even if the predecessor *B* is showing an increase resp. decrease. On the other hand, Constraint 2 forbids *D* to increase or decrease, if all predecessors are labeled as 0-change.

### From local to global reasoning

**Fig. 4:**
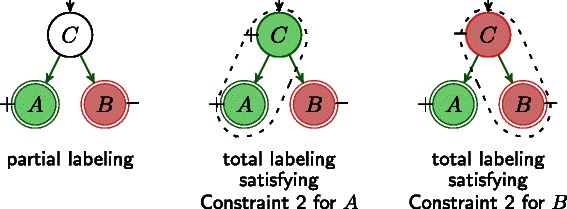
Example for an influence graph with partial labeling, which is locally consistent for *A* and *B*, but globally inconsistent because there exist no single total labeling satisfying Constraint 2 for *A* and *B*

While there might exist several total labelings that satisfy the local constraints for *some* nodes we are interested in checking global consistency, where a total labeling exists such that the local constraints are satisfied for *all* nodes. In Fig. 4, we illustrate an IG together with a partial labeling which is locally consistent but globally inconsistent. In other words, there exist two total labelings such that the *local consistency rules* (Constraints 1, 2 and 3) are satisfied, for either *A* or *B*, but there exists no single total labeling that satisfies these constraints for all nodes.

We use the previously defined constraints to define the following global consistency notions.

#### **Consistency Notion1** (weak propagation, WP).

We call an IG and an experimental profile (*S*,*I*,*μ*) consistent under *weak propagation* (WP), iff there exists a total labeling *μ* ^*t*^ such that Constraint 1 and 2 are satisfied for all nodes.

#### **Consistency Notion2** (strong propagation, SP).

We call an IG and an experimental profile (*S*,*I*,*μ*) consistent under *strong propagation* (SP), iff there exists a total labeling *μ* ^*t*^ such that Constraints 1, 2 and 3 are satisfied for all nodes.

**Fig. 5:**
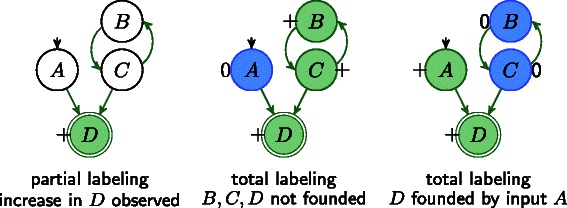
Example for an influence graph with partial labeling, which can be explained either by self activation in *B* and *C* or by the input node *A*

Further, we introduce here a new *global constraint* to ensure that every node change is justified by a chain of influences that can be traced back to an (perturbed) input node. This natural constraint is especially useful to forbid self-justification of changes via positive feedback loops (see Fig. 5).

#### **Constraint4** (a change must be founded in an input).

Let (*V*,*E*,*σ*) be an IG, (*S*,*I*,*μ*) an experimental profile, *μ* ^*t*^:*V*→{+,−,0} be a total labeling, and *i*∈*V* a node with *μ* ^*t*^(*i*)∈{+,−}.

Then *μ* ^*t*^ satisfies Constraint 4 for node *i* if either *i* is an input node *i*∈*I*, or there exist a path (*v*_0_,…,*v*_*k*_) in *E* with *v*_0_∈*I*, *v*_*k*_=*i* and *μ* ^*t*^(*v*_*n*−1_)*σ*(*v*_*n*−1_,*v*_*n*_)=*μ* ^*t*^(*v*_*n*_) for all *n*=1…*k*.

In Fig. 5, we illustrate an IG with a partial labeling (left) and two total labelings (middle and right) derived from the partial one. Both total labelings satisfy the local propagation rules (Constraints 2, 3), but only the second total labeling satisfies the global propagation rule (Constraint 4). While the first labeling suggests a self-sustained increase in *B* and *C* as explanation for the increase in *D*, the second labeling hints to an increase in the input node *A*. Using Constraint 4 we can avoid manual removal of positive feedback loops as done in previous studies [17].

We combine the new constraint with previously defined constraints into the following consistency notions.

#### **Consistency Notion3** (founded weak propagation, FWP).

We call an IG and an experimental profile (*S*,*I*,*μ*) consistent under *founded weak propagation* (FWP), iff there exists a total labeling *μ* ^*t*^ such that Constraints 1, 2 and 4 are satisfied for all nodes.

#### **Consistency Notion4** (founded strong propagation, FSP).

We call an IG and an experimental profile (*S*,*I*,*μ*) consistent under *founded strong propagation* (FSP), iff there exists a total labeling *μ* ^*t*^ such that Constraints 1, 2, 3, and 4 are satisfied for all nodes.

### Consistency checking

**Fig. 6:**
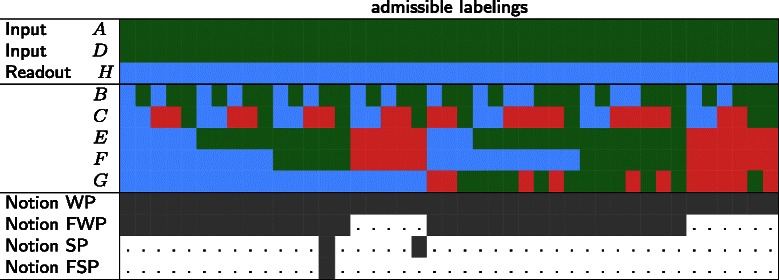
Consistent total labelings of the example in Fig. 1 under different consistency notions. The consistent labelings under all four consistency notions are fully displayed, a grey cell indicates that the labeling above is consistent and a white cell with “.” means that it is not a consistent labeling

We can now apply the previously defined consistency notions to enumerate consistent total labelings and to verify the consistency of network and observation data for a given experimental profile. We consider an IG consistent with an experimental profile (*S*,*I*,*C*) if there exists at least one consistent total labeling (consistent with respect to the chosen Notion WP, SP, FWP or FSP). Consider Fig. 6 which shows the total labelings of the IG in Fig. 1 consistent with an example experimental profile (*A* and *D* were increased resulting in a measured 0-change in *H*) under the different consistency notions. Note that the notions become more strict, accepting less labelings as consistent and therefore excluding certain system behaviors. The set of admissible labeling under SP is a subset of the admissible labelings under WP and the set of admissible labeling under FSP is a subset of the admissible labelings under SP. Further, one can see that Constraint 4 excludes all labelings where *E* and *F* decrease. This behavior does not satisfy Constraint 4, as it is only possible by mutual inhibition using the positive loop between *E* and *F*, which is not founded in an input.

#### Predictions under consistency

The consistency check of network and experimental data is the first analysis that is performed with the sign consistency approach. If network and data are consistent the sign consistency approach can be used to predict the behavior of unmeasured entities in the network. This can also be used to predict the outcome of a planed experiment and reversely to plan an experiment that should result in a specific desired behavior. In the sign consistency approach, each consistent labeling represents an admissible behavior of the system. We call a statement that holds in all admissible behaviors under the given consistency notion a *prediction*. If parts of the system act the same in all admissible behaviors this can be predicted. We can predict the following types of behaviors in our systems. We predict that a species *increases* + (resp. *decreases* −, does *not change* 0) if it increases (resp. decreases, does not change) in all admissible labelings. We call these strong predictions, because the possible behaviors of a species are reduced to exactly one. Further, we can predict that a species does not increase (resp. does not decrease, does change) if it does not increase (resp. not decrease, does change) in all admissible labelings. Therefore, we can also predict *weak increase* ***⊕***, when a species does not decrease, but increases in at least one admissible behavior, and does not change in another admissible behavior. Likewise, we predict *weak decrease* ***⊖*** when a species does not increase, but decreases in at least one admissible behavior, and does not change in another. Finally, we predict *change* ± when a species does always change, it increases in at least one admissible behavior and decreases in another. We call ***⊕***, ***⊖***, and ± weak predictions because one possible behavior is excluded while one degree of freedom is left.

Formally, for a set *V* of nodes in our network and the set *M* of labelings consistent with our experimental profile, we define the prediction function *p* *r* *e* *d*:*V*→{+,−,0,***⊕***,***⊖***,±, no } as follows:

$${} pred(x) \,=\,\left \{ \begin{array}{cll} + & |~ \forall \mu \in M: \mu(x)= +, \\ - & |~ \forall \mu \in M: \mu(x)= -,\\ 0 & |~ \forall \mu \in M: \mu(x)= 0, \\ \\ \boldsymbol{\oplus} & |~ \forall \mu \in M: \mu(x)\neq -, & \exists \mu \!\in\! M: \mu(x)= +,\\ & & \exists \mu \!\in\! M: \mu(x)= 0,\\ \boldsymbol{\ominus} & |~ \forall \mu \in M: \mu(x)\neq +, & \exists \mu \!\in\! M: \mu(x)= -,\\ & & \exists \mu \!\in\! M: \mu(x)= 0,\\ \pm & |~ \forall \mu \in M: \mu(x)\neq 0, & \exists \mu \!\in\! M: \mu(x)= +,\\ & & \exists \mu \!\in\! M: \mu(x)= -,\\ \text{no} & |~ else. \end{array} \right. $$

#### Recovery rate and precision

In Table 1, we show the predictions for the example given in Fig. 1. One can see that the more constrained consistency notions yield smaller sets of admissible labelings and a higher *recovery rate* (for how many unmeasured species can predictions be obtained). In the systematic comparison of the consistency notions based on real experimental data we not only consider recovery rate but also prediction *precision* (true positives/(true positives + false positives)). A strong prediction (+ / −/0) will be a true positive if it has a certain measurement with the same value (+/ −/0). A weak prediction ***⊕*** (resp. ***⊖*** and ±) will be a true positive if it has a certain measurement + or 0 (resp. − or 0 and + or −). Reversely, a prediction will be a false positive if has a certain measurement value with a contradictory value +.

**Table 1:** Predictions for the example in Fig. 1 derived from the admissible behaviors in Fig. 6

	*B*	*C*	*E*	*F*	*G*
Notion WP	***⊕***	no	no	no	no
Notion FWP	***⊕***	no	***⊕***	***⊕***	no
Notion SP	+	±	±	±	0
Notion FSP	+	+	+	+	0

The **no** means that the node can have any value of {+, 0, −} which means that pratically no prediction is possible

### Repairing inconsistent networks and data

If network and data are inconsistent the natural question arising is how to repair networks and/or data, that is, how to modify network and/or data in order to re-establish their mutual consistency. A major challenge lies in the range of possible repair operations, since an inconsistency can be explained by missing interactions or inaccurate information in a network as well as by measurement errors. The sign consistency approach can be used to determine a set of repair operations that are suitable to restore consistency. Typically, plenty of suitable repair operations are possible, in particular, if multiple repair operations are admitted. However, one usually is only interested in repairs that make few changes on the model and/or data. These minimal repair sets cannot only be used for hypotheses generation (e.g., which data might be questionable or which edges might be missing or inactive) but as a quantitative measure for the fitness of model and data. Also note that once consistency is re-established, network and data can again be used for predicting behaviors of unmeasured entities.

In [17], four repair operations were introduced; two of them for single experiments (SCEN-FIT, Minimal Correction Sets (MCoS)) and two for multiple experiments (OPT-SUBGRAPH, OPT-GRAPH). The latter two are computationally more demanding as they seek to optimize the whole network structure based on many perturbation experiments. SCEN-FIT, as explained in detail in the Additional file 1, seeks to find a consistent node labeling that is closest to the given measurements and can thus help to identify inconsistencies between network and dataset. Herein we will focus on MCoS and thus deal with analysis of single experiments. This is motivated by our application example where we indeed have multiple experiments (105) but where the number of experiments is low compared to the number of edges and nodes in the network (1646) disabling a meaningful network structure optimization. However, we note here that our extended notion FSP can be straighforwardly applied to these repair operations as well.

#### Minimal Correction Sets (MCoS)

**Fig. 7:**
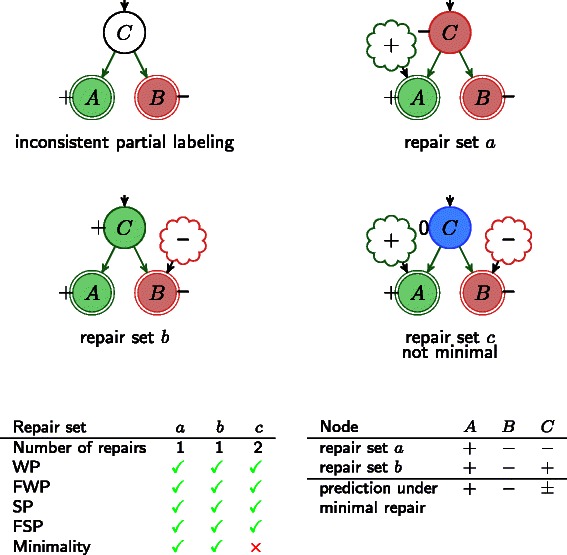
Repair by adding signed influences example (Minimal Correction Sets - MCoS). There exist three alternative repair sets: repair set *a* adds a positive influence to *A* and repair set *b* includes a negative influence on *B*, repair set *c* includes a positive influence on *A* and a negative influence on *B*. Repair sets *a* and *b* are minimal containing only one repair, repair set *c* is not minimal having two repairs. Looking at the intersection of the labelings under minimal repairs, we can conclude that *C* is either responsible for an increase in *A* or a decrease in *B*. We can therefore exclude a labeling of *C* with 0, we can predict: *p* *r* *e* *d*(*C*)=±

To resolve inconsistencies one may add new influences to the model if the later is considered to be potentially incomplete (which is often the case in practice). Adding an influence can be used to indicate missing (unknown) regulations or oscillations of regulators that would explain the (topology-inconsistent) measurements. We use minimal correction sets (MCoS) as defined in [17] as minimal sets of new signed (positive or negative) input influences that restore consistency of model and data. MCoS are defined as signed influences and are specific for a single experiment; they might be incompatible with other experiments. Note that every inconsistency can be repaired by adding a new influence. Therefore, adding influences is always suited to restore consistency. Also the MCoS can be interpreted as a measure of consistency of model and data. Compared to SCEN-FIT, MCoS yields always a smaller or equal number of repairs. Therefore we define the inconsistency-index of a network with respect to data as (MCoS/number of observations in the experiment). Figure 7 illustrates how repair through addition of influences works.

#### Prediction under minimal repair

Due to the capability of repairing, the sign consistency approach enables prediction even if model and data are mutually inconsistent. Predictions under minimal repair are obtained from the identification of consequences shared by all consistent labelings under all possible minimal repairs. Note that this approach although it confines to minimal repairs following the law of parsimony, does not favor any of the possible minimal repairs but only considers a statement a prediction if it holds under every minimal repair.

### Software

The different consistency notions as well as the methods for consistency checking and quantification, prediction, and all data and network repair operations were implemented in an open source application iggy [24]. iggy uses ASP [23] as logical modeling and constraint solving paradigm, it is part of the BioASP software collection and can easily be installed via the python package index (PyPI). ASP is used to model problems from NP and provides state-of-the-art solvers. In particular, we use the solver clasp [25] via the pyasp [26] package. On an AMD Opteron 6168 1.9 GHz with 96 GB RAM, given a network with 1646 nodes and 4277 edges our software needs ≈20 min to compute the predictions under minimal repair (MCos) for the unmeasured species of 105 experiment data sets each containing 1392 measurements. For further information visit http://bioasp.github.io/iggy.

## Results and discussion

To investigate the suitability of the different consistency notions, we used the gene regulatory network of *Escherichia coli* and confronted it with Microarray data. The network was obtained from RegulonDB [27], version 8.3 in october 2013, and we focused on its biggest weakly connected component which is composed of 1646 nodes and 4277 edges and covers 94 *%* of the nodes of the full RegulonDB network. Unsigned edges are treated as two parallel edges with opposite signs. The data refers to the microarray log ratio expression of 3607 genes measured under 240 different stress conditions in *E. coli* published in [28]. We chose 105 of 240 experiments which can be interpreted as steady state shift experiments and 1392 of the 3607 genes which occur in the RegulonDB network. Since the input nodes for the stress condition experiments are unknown, we simply defined all nodes without predecessors as inputs.

The GEO/GSE codes for the used experiments are listed in the Additional file 1. The microarray data was discretized as described in the Methods Section using the typical thresholds: *t*_1_=−2, *t*_2_=−0.01, *t*_3_=0.01, *t*_4_=2, to generate the constraints that restrict the labeling *μ* for the nodes measured in the experimental profile.

To evaluate the influence of the minimal correction sets (MCoS) and to investigate the suitability of the different consistency notions to predict the behavior of unobserved entities in a regulatory network, we performed a cross-validation using the *E. coli* data.

### Quality of regulatory network when confronted to the expression profiles

**Fig. 8:**
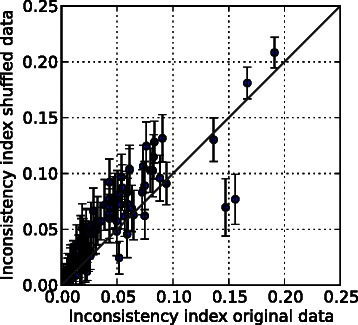
Inconsistency index of the 105 experiments. The x-axis shows the inconsistency index of the original experiments and the y-axis the average inconsistency of the shuffled experiments. The error bars indicate the standard deviation of the inconsistency index among 100 shuffled samples

As a first step, we assess the quality of network and data by comparing it to randomized data. We generated 100 randomized datasets for each real experiment by shuffling the observed signs among the observed nodes; but preserving the sign distribution for each dataset. We then computed for real and randomized data the inconsistency index which is defined as the quotient of the number of minimal corrections (MCoS) to restore consistency (under notion FSP) divided by the number of observations in the experiment. Then we computed the Wilcoxon signed-rank test to assess whether the population means of the two samples differ. The obtained p-value of 2.0497*e*-11 indicates a highly significant difference of real and randomized data, suggesting that the real data are more (sign-) consistent with the network topology than random data. Figure 8 shows the *inconsistency index* for real and randomized data for each experiment. We can see that the real *E. coli* dataset exhibits a significantly lower inconsistency index than the randomized data.

**Fig. 9:**
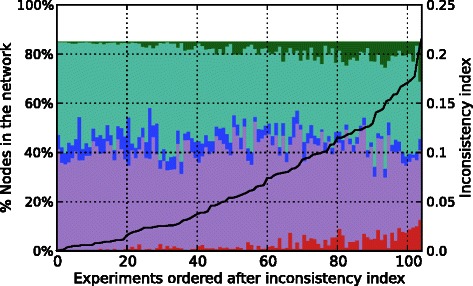
Distribution of observed signs in the experimental data. The x-axis shows the 105 experiments ordered after their inconsistency index represented by the black line, getting less consistent from left to right. The left y-axis quantifies the cumulative percentage of nodes in the network which were measured. The signs are represented by the following colors: − (red), $\triangledown $ (purple), 0 (blue), $\vartriangle $ (turquoise), and + (green)

Figure 9 shows the distribution of the measured signs in the experimental data revealing that the data tends to be less consistent if more +/- are contained.

### Predictions under the different consistency notions

To investigate the suitability of the different consistency notions to predict the behavior of unobserved entities in a regulatory network, we performed a cross-validation using the *E. coli* data. While other validation methods exist, we decided to use cross-validation as a model validation technique because it allows us to assess how the results of the approach will generalize to independent datasets. To set up cross-validation, we created for each experiment 100 samples each containing a random 10 *%* share of the measurements. We then confronted the *E. coli* network with these samples, determined the minimal corrections necessary to restore consistency, and computed the predictions that hold under all minimal correction sets.

In Table 2 one can see the distribution of the +, −, 0 and weak predictions as well as how the precision of the different types of predictions varies among the different notions (WP is similar to FWP see Additional file 1). With the different consistency notions we were on average able to compute behavior predictions for up to 69 *%* of the remaining nodes in the network, for which no measurement was given. One can observe that the share of nodes with predictions increases drastically with notion SP and even further with FSP, mainly through an increased prediction of 0-change behaviors.

**Table 2:** Average % of unobserved nodes that have predictions of a particular behavior, their information gain and the precision for these predictions under the different notions giving 10 *%* or 50 *%* of the *E. coli* expression measurements as input. In the last major column (“all predictions”) the rows “% of unobserved nodes” quantify the overall recovery rates

Prediction	+ / −			0			Weak predictions ***⊕***/ ***⊖***/ ±			All predictions		
Notion	FWP	SP	FSP	FWP	SP	FSP	FWP	SP	FSP	FWP	SP	FSP
Obtained using 10 % of the measurements as input.												
% of unobserved nodes	0.13 %	2.60 %	2.91 %	1.19 %	46.43 %	53.64 %	7.74 %	12.99 %	13.14 %	9.06 %	62.03 %	69.70 %
Information gain	0.13 %	2.60 %	2.91 %	1.19 %	46.43 %	53.64 %	2.86 %	4.80 %	4.85 %	4.17 %	53.83 %	61.41 %
Precision of prediction	29.02 %	56.28 %	54.50 %	82.16 %	70.69 %	71.66 %	80.94 %	85.19 %	85.14 %	80.03 %	72.97 %	73.24 %
***P*** *-value*										**0.2791**	**1.0389e-08**	**8.0897e-10**
Obtained using 50 % of the measurements as input.												
% of unobserved nodes	0.48 %	3.06 %	3.08 %	4.84 %	67.93 %	72.30 %	28.99 %	7.27 %	7.07 %	34.31 %	78.26 %	82.45 %
Information gain	0.48 %	3.06 %	3.08 %	4.84 %	67.93 %	72.30 %	10.70 %	2.68 %	2.61 %	16.02 %	73.67 %	77.99 %
Precision of prediction	24.10 %	62.16 %	62.14 %	82.79 %	70.22 %	70.97 %	82.44 %	86.75 %	86.80 %	81.33 %	71.10 %	71.57 %
***P*** *-value*										**0.5**	**2.9782e-09**	**2.0785e-10**

The different types of predictions contain different amount of informations. A weak prediction gives less information than a strong prediction because it discards only one out of three possible labels. Hence, the 69 *%* of nodes with prediction does not equal 69 *%* of information gained. Therefore, we also computed the information gain given by these predictions. For *n* unconstrained nodes, for which no measurements are taken into account, 3^*n*^ possible behaviors exist, for *k* nodes with strong predictions the possible behaviors can be restricted to just 1, and for *l* nodes with weak predictions remain still 2^*l*^ possible behaviors, for *m* nodes without predictions remain still 3^*m*^ possible behaviors, and the overall information gain can then be expressed as (*l* *o* *g*(3^*n*^)−*l* *o* *g*(1^*k*^+2^*l*^+3^*m*^)/*l* *o* *g*(3^*n*^)). In our experiments we observed an average information gain up to 61 *%* for the nodes for which no measurements had been taken into account. For more information on how to compute the information gain we refer to the Additional file 1.

To validate the quality of the predictions (obtained from 10 *%* of the data), we compared them with the validation data (the remaining 90 *%* of measurements). For the nodes where a prediction and validation data was available, we compared both. We obtained on average precisions that range from 73 *%* to 80 *%*. Overall, SP and FSP allow us to make predictions for a much bigger part of the network, resulting in a much higher information gain with only a slightly decreased precision, and for + and − predictions with a significant higher precision than FWP. In Section 5 of the Additional file 1 we plot the detailed recovery and precisions per experiment for notions FWP and FSP.

To test the influence of the number of measurements on recovery rate and precision, we also created a dataset with 50 *%* and 75 *%* (see Additional file 1: Table S3) of the measurements. Compared to the results with 10 *%* the overall recovery rate increases up to 82 *%* (FSP). This is due to the fact that the increased amount of data helps to put more constraints on the systems behavior. For notion SP and FSP the number of weak predictions drops slightly because many of them become strong predictions. The precision of +, − and weak predictions benefits from the richer datasets under notion SP and FSP, while the precision of 0-change decreases only slightly.

Weak predictions easily have higher precisions, because they have a bigger chance to be true positives. To validate that the precisions obtained in our test case are indeed meaningful, we tested our approach on a randomized dataset. We could verify that the predictions from randomized data have less precision than the predictions obtained from the real data (see Additional file 1: Table S3),especially for notions SP and FSP. Accordingly the p-values shown in Table 2 indicate a high significance that the predictions made by SP and, even more pronounced, by FSP are better than random.

**Fig. 10:**
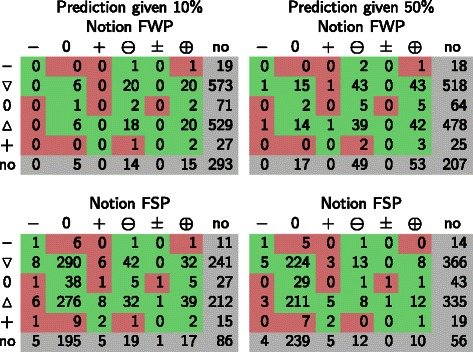
Confusion matrices for predicted behaviors under each notion given 10*%* (left) 50*%* (right) of the data. The columns indicate the predicted behavior and the rows the measured behavior. Given are the average numbers of nodes predicted to change as indicated by the column and measured in the experiment as indicated by the row with respect to the total number of measured predictions. Predictions that are consistent with the measurement are in green and confusions in red. Gray fields denote either predictions that could not be verified because no validation data was available or nodes for which validation data was available but no predictions had been made

These results show that the strong-propagation notions (SP and FSP) are the most pertinent choice to explain gene expression shifts within the *E. coli* transcriptional network. Using FSP we predict with high precision that 53 *%* to 72 *%* of the network remains unaltered (0-change). Understanding the differentially expressed network regions becomes more delicate, since the precision remains on average 54 *%* to 62 *%* which, however, is still significantly higher than for notion FWP. Nevertheless, 48 *%* of the experiments had a precision above 75 *%* for up- or down-regulation (strong) predictions when considering a dataset with 50 % of the measures. Note, that the notion of precision changes its conclusiveness when applied to incompletely determined predictions. Thus, we use confusion matrices as an alternative representation to illustrates the performance of our prediction method. Here one can see that for uncertain observations, relatively few strong predictions are confused (see Fig. 10). Therefore, wrong predictions may be related to the choice of the discretization thresholds and that a single threshold was chosen for all genes.

## Conclusion

We presented a unified framework to express different notions of sign consistency on interaction graphs. A refined methodology for data discretization into five values allows the consideration of uncertainties in experimental profiles. Within this framework we introduced a new constraint to filter undesired self-fulfilled regulations that result from positive feedback loops. Finally, our extended prediction method considers not only strong (unique value) but additionally weak (multiple admissible values) predictions, enlarging the predictive power of the approach.

We evaluated our framework by confronting the full RegulonDB network with 105 experimental gene-expression profiles. Our cross-validation results obtained when choosing 10 *%* of the initial dataset show that the overall precision of the methods ranges from 72 *%* to 80 *%*. The precision of the FSP notion has a much higher and significant p-value. With its increased precision and recovery, FSP appears to be the superior notion.

We expect that the information gain is in general higher for datasets from (typically smaller) signaling networks (see e.g. [17]). This might be due to the fact that in the stress experiments considered here the (perturbed) inputs of the gene regulatory network were unknown which poses less constraints than in signaling networks with normally well-defined signal inputs (given by the applied ligands, inhibitors etc.).

Our method requires a careful selection of discretization thresholds. Therefore, we performed a detailed sensitivity analysis on a wide range of the discretization thresholds (see Additional file 1: Section 4). The analysis shows that there is a relatively small sensitivity of the results (precision, information gain) w.r.t. the chosen thresholds. We also discuss further aspects of threshold selection in the Additional file 1.

There is a relationship between the concept of sign consistency and the dependency matrix (discussed in more detail in [17]). The notion of the dependency matrix was originally introduced in [4] and has been used in several studies for checking consistency between signaling network topologies and experimental data from stimulus-response experiments, (e.g., [5, 29]). In fact, the dependency matrix can be seen as another sign consistency notion which is more relaxed than SP or FSP (what might still be useful, e.g. when analyzing transient instead of steady-state responses). Since additional propagation rules are straightforward to implement in the framework presented herein, other sign consistency notions, including the dependency matrix or those that pose different constraints for 0-changes, could be considered as well. Overall, our work enhances the flexibility and power of the sign consistency approach for the prediction of the behavior of signaling and gene regulatory networks and, more generally, for the validation and inference of these networks.

## Additional file

Additional file 1 **Supplementary.** Contains the following supplementary material. Explanation of SCEN-FIT. Explanation of uncertain observations. Information gain by predictions in the sign consistency approach. Sensitivity analysis - Choosing the thresholds for discretization. Recovery and precision for *E. coli* cross-validation experiments. GEO/GSEcodes for the experiments used. (PDF 1218 kb)

## Abbreviations

IG: Influence graph
ILP: Integer linear programming
ASP: Answer set programming
WP: Weak propagation
SP: Strong propagation
FWP: Founded weak propagation
FSP: Founded strong propagation
