# Supplementary material for: Extended notions of sign consistency to relate experimental data to signaling and regulatory network topologies
Source: BMC Bioinformatics. 2015 Oct 28;16:345. doi: 10.1186/s12859-015-0733-7 (PMC4625540; doi:10.1186/s12859-015-0733-7)
Supplement: Supplementary file 1 — Supplementary. Contains the following supplementary material. Explanation of SCEN-FIT. Explanation of uncertain observations. Information gain by predictions in the sign consistency approach. Sensitivity analysis - Choosing the thresholds for discretization. Recovery and precision for E. coli cross-validation experiments. GEO/GSEcodes for the experiments used. (PDF 1218 kb) [file 12859_2015_733_MOESM1_ESM.pdf]

Supplements

Extended Notions of Sign Consistency to Relate Experimental Data to Signaling and Regulatory Network Topologies

Sven Thiele, Luca Cerone, Julio Saez-Rodriguez, Anne Siegel, Carito Guziolowski and Steffen Klamt

1 SCEN-FIT

SCEN-FIT seeks to find a consistent node labeling that is closest to the given measurements. SCEN-FIT can thus help to identify inconsistencies between network and dataset and to detect potential measurement errors. If a dataset is not consistent (i.e. no suitable node labeling containing the measurements can be found) consistency can be restored by revising an inconsistent labeling $\mu : V \to \{+, -, 0\}$ to a consistent labeling $\mu^c : V \to \{+, -, 0\}$. The fitting error is defined as the cost for restoring consistency, the absolute difference $\sum_{i \in V} |\mu(i) - \mu^c(i)|$ between observed labeling μ and the closest consistent labeling μ^c (SCEN-FIT). Note that SCEN-FIT revisions do not revise the labeling of input nodes as these are considered under control of the experimentalist.

In the ideal case where data and network are consistent none of the observed changes has to be revised and the fitting error is 0. Note that every IG has at least one consistent labeling (labeling all nodes zero). Even if the labels of the input nodes are fixed to non-0 values, at least one consistent labeling exists. Therefore, revising observed changes is always suited to restore consistency. In Figure 1 we illustrate how repair through revision of observations works.

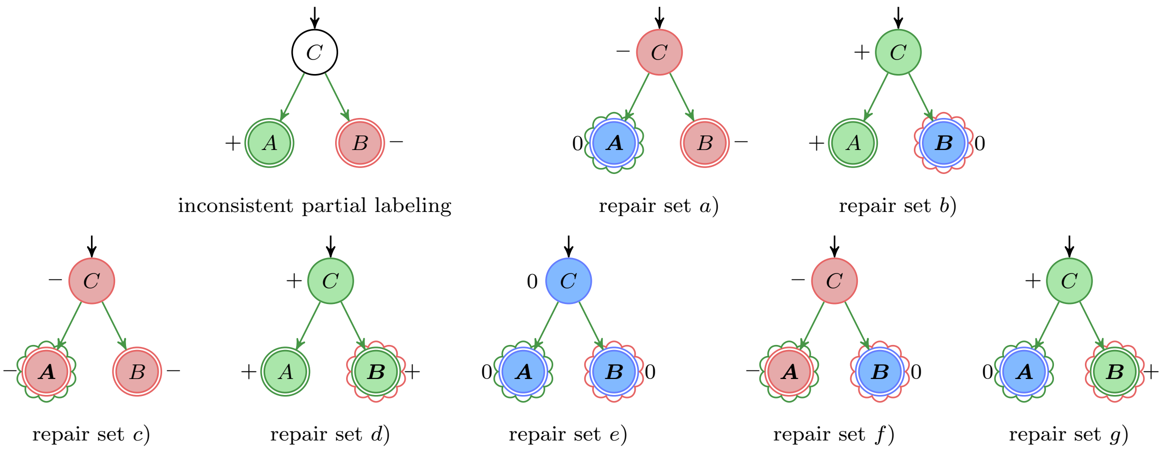

Repair set	(a)	(b)	(c)	(d)	(e)	(f)	(g)
Cost (number of repairs)	1	1	2	2	2	3	3
Weak consistency	✓	✓	✓	✓	✓	✓	✓
Filtered weak consistency	✓	✓	✓	✓	✓	✓	✓
Strong consistency	×	×	✓	✓	✓	×	×
Filtered strong consistency	×	×	✓	✓	✓	×	×
Minimality	minimal for weak consistency		minimal for strong consistency			not minimal	

Node	A	B	C
repair set (a)	0	-	-
repair set (b)	$+$	0	$+$
prediction under minimal repair for weak consistency	$\oplus$	$\ominus$	$\pm$

Node	A	B	C
repair set (c)	-	-	-
repair set (d)	$+$	$+$	$+$
repair set (e)	0	0	0
prediction under minimal repair for strong consistency	×	×	×

Supplementary Figure 1: Repair by SCEN-FIT. The picture shows 7 alternative repair sets for the inconsistent labeling shown top left. The repair sets a and b have costs of 1, repair set a revises the labeling of A from $+$ to 0 and repair set b revises B from $-$ to 0. They both restore consistency regarding Notion WP and WFP but not regarding Notion SP. The repairs sets c-e have costs of 2 and restore consistency regarding all Notions (WP,WFP,SP, and SFP). Repairs set c revises the labeling of A from $+$ to $-$, repairs set d revises B from $-$ to $+$, and repair set e revises both A and B to 0. The repairs sets f and g have costs of 3 and restore consistency regarding Notion WP but not Notion SP. Repair set f revises A to $-$ and B to 0, and repair set g revises A to 0 and B to $+$. Repair sets a and b are minimal regarding Notion WP. Therefore under Notion WP and under minimal repair we predict: $pred(C) = \pm$, $pred(A) = \oplus$, $pred(B) = \ominus$. Repair sets c-e are minimal regarding Notions SP and SFP. In this case the alternative repairs remove all constraints on the system and we predict nothing. Repair sets f and g restore consistency under Notion WP but are not minimal.

2 Uncertain observations

Uncertain observations restrict the possible labelings of a node to two out of three labels (+, 0, −). An uncertain-increase (△) allows + and 0 and an uncertain-decrease (▽) allows − and 0.

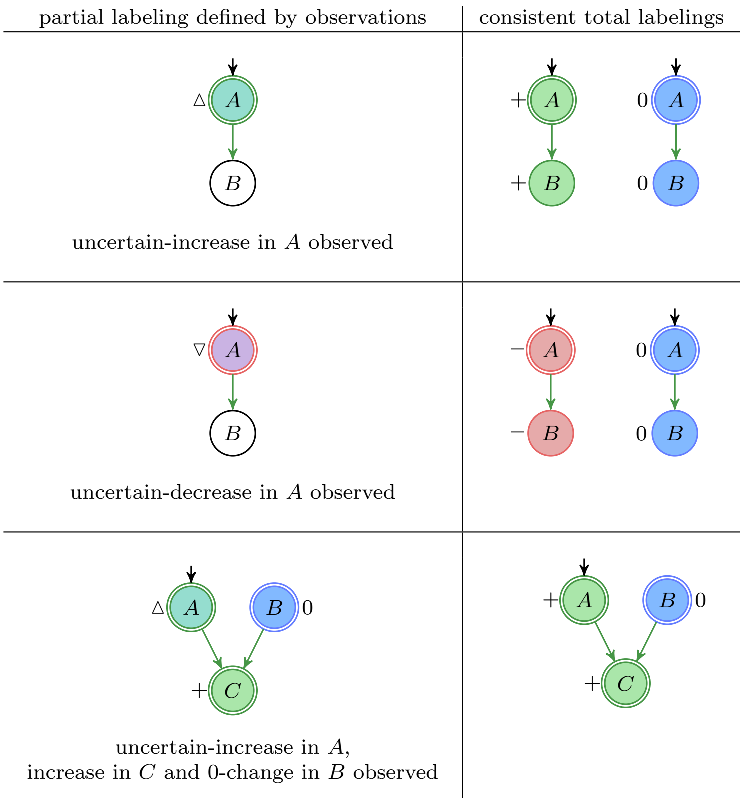

Supplementary Table 1: Example for an influence graphs with partial labelings containing uncertain observations, and the corresponding consistent total labelings.

3 Information gain by predictions in the sign consistency approach

We define the information gain achieved by the constraints of our consistency notions as the entropy difference of our system with vs. without constraints.

The entropy $H(X)$ of a random variable X with possible values $\{x_1, \ldots, x_k\}$ and a probability function $P(x_i)$ is defined as

$$H(X) = -\sum_{i=1}^{k} P(x_i) \times log_2 P(x_i).$$

The unconstrained graph A graph with n nodes where each node can take on of three values $\{-, 0, +\}$ can be represented as a random variable X_{graph} with 3^n possible values. Assuming an equal probability for each state the entropy $H(X_{graph})$ is defined as follows:

$$H(X_{graph}) = log_2(3^n).$$

For the *E. coli* graph with $n = 1646$ this would be

$$log_2(3^{1646}) = 2953\ bit.$$

The graph + measurements Measurements can be used to limit the possible behaviors of the system. If we use the discretization approach that is explained in the paper we can distinguish three kind of nodes. We have sm nodes with certain measurements +, − and 0 (they have one fixed value), wm nodes with uncertain measurements can (each can have one of two values), and um unmeasured nodes (each can have one of three values).

Including these constraints, the entropy $H(X_{graph+obs})$ of our system is defined as follows:

$$H(X_{graph+obs}) = log_2(1^{sm} \times 2^{wm} \times 3^{um}).$$

For the *E. coli* example with 10% of the data we have $sm \approx 13$, $wm \approx 119$, and $um \approx 1514$. The entropy would be

$$log_2(1^{13} \times 2^{119} \times 3^{1514}) \approx 2519\ bit.$$

For the *E. coli* example with 50% of the data we have $sm \approx 64$, $wm \approx 597$, and $um \approx 985$. The entropy would be

$$log_2(1^{64} \times 2^{597} \times 3^{985}) \approx 2158\ bit.$$

For the *E. coli* example with 100% of the data we have $sm \approx 127$, $wm \approx 1195$, and $um \approx 324$. The entropy would be

$$log_2(1^{127} \times 2^{1195} \times 3^{324}) \approx 1709\ bit.$$

The graph + measurements + predictions Sign consistency constraints reduce the possible system states. This information gain is expressed by the predictions. We can therefore use the predictions to further distinguish the set of nodes into sp nodes without measurement and a strong prediction (they have one fixed value), wp nodes without measurement and a weak prediction (each can have one of two values), and up nodes without measurements and without prediction (each can have one of three values). Including the predictions, the entropy $H(X_{graph+obs+pred})$ of our system is defined as follows:

$$H(X_{graph+obs+pred}) = log_2(1^{sm+sp} \times 2^{wm+wp} \times 3^{up}).$$

Note that for simplicity we only consider predictions of unmeasured nodes, but the constraints can also restrict the possible values for nodes with uncertain measurements and discard some value combinations.

For the *E. coli* example with 10% of the data and notion FWP we have $sp \approx 17$, $wp \approx 116$, and $up \approx 1318$. The entropy would be

$$log_2(1^{13+17} \times 2^{119+116} \times 3^{1318}) \approx 2314\ bit.$$

For the *E. coli* example with 50% of the data and notion FWP we have $sp \approx 52$, $wp \approx 287$, and $up \approx 646$. The entropy would be

$$log_2(1^{64+52} \times 2^{597+287} \times 3^{646}) \approx 1908\ bit.$$

For the *E. coli* example with 10% of the data and notion FSP we have $sp \approx 858$, $wp \approx 199$, and $up \approx 457$. The entropy would be

$$log_2(1^{13+858} \times 2^{119+199} \times 3^{457}) \approx 1042\ bit.$$

For the *E. coli* example with 50% of the data and notion FSP we have $sp \approx 743$, $wp \approx 69$, and $up \approx 173$. The entropy would be

$$log_2(1^{64+743} \times 2^{597+69} \times 3^{173}) \approx 940\ bit.$$

Information gain The information gain is defined as follows:

$$(H(X_{graph+obs}) - H(X_{graph+obs+pred}))/H(X_{graph+obs})$$

.

4 Sensitivity analysis - Choosing the thresholds for discretization

In this Section, we explain some points that have to be considered when choosing the thresholds. Over all experiments used in this paper we have in total 146160 measurements. 4 thresholds (t_1, t_2, t_3, t_4) are used to discretize the data (see main manuscript). Due to the symmetrie in our measurement distribution we choose $t_3 = -t_2$ and $t_4 = -t_1$. All values smaller than t_1 are discretized as decrease, all values greater than t_4 are discretized as increase, all values between t_2 and t_3 are discretized as 0-change, and values between t_1 and t_2 (resp. between t_3 and t_4) are discretized as uncertain-decrease (resp. uncertain-increase).

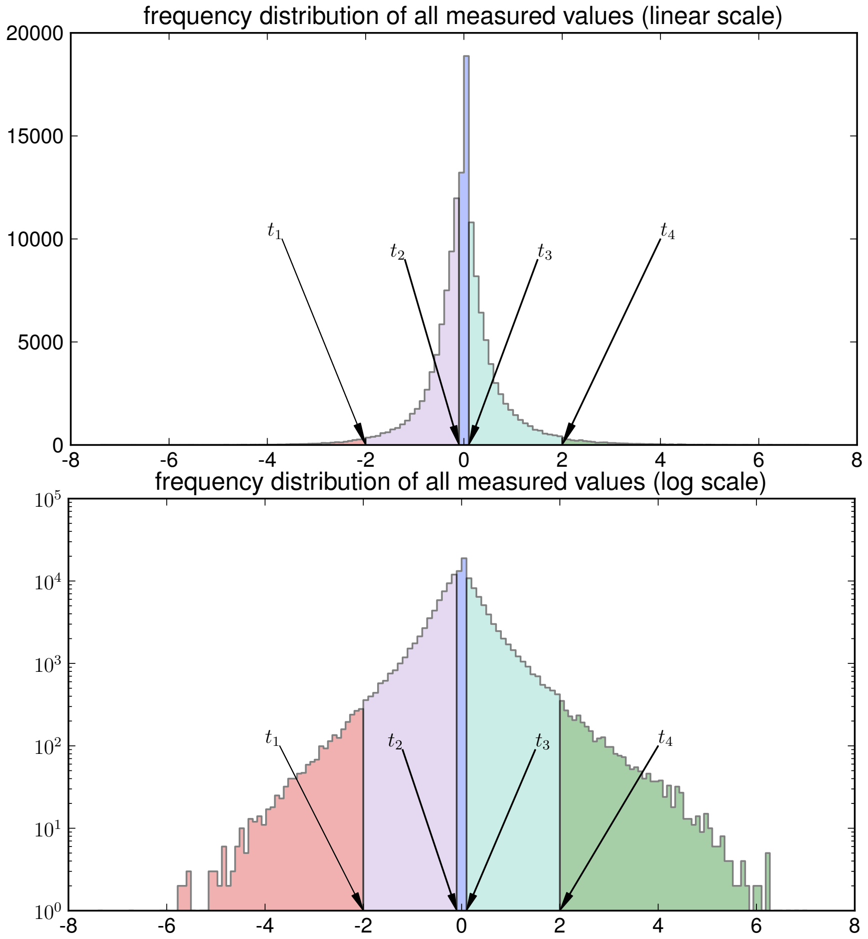

Supplementary Figure 2: Frequency distribution of the measured log ratio changes over all 146160 data points, on top using a linear scale and below using a logarithmic scale.

The frequency distribution of all the measurements in our data set (see Supplementary Figure 2), shows that the data is symmetric and that most of the measurements are close to 0. Therefore, a small change in the t_2 and t_3 threshold affects most of the measurements, while changes in the t_1 and t_4 threshold affects only relatively few measurements. More precisely, increasing the absolute values of t_2 and t_3 leads to many more measurements classified as 0-changes. While decreasing the absolute values of t_1 and t_4 only leads to relatively few more measurements classified as increase/decrease. Therefore, the thresholds $t_3 = -t_2 = 0.01$ and $t_4 = -t_1 = 2.0$ are a conservative choice as they classify many measurements as uncertain.

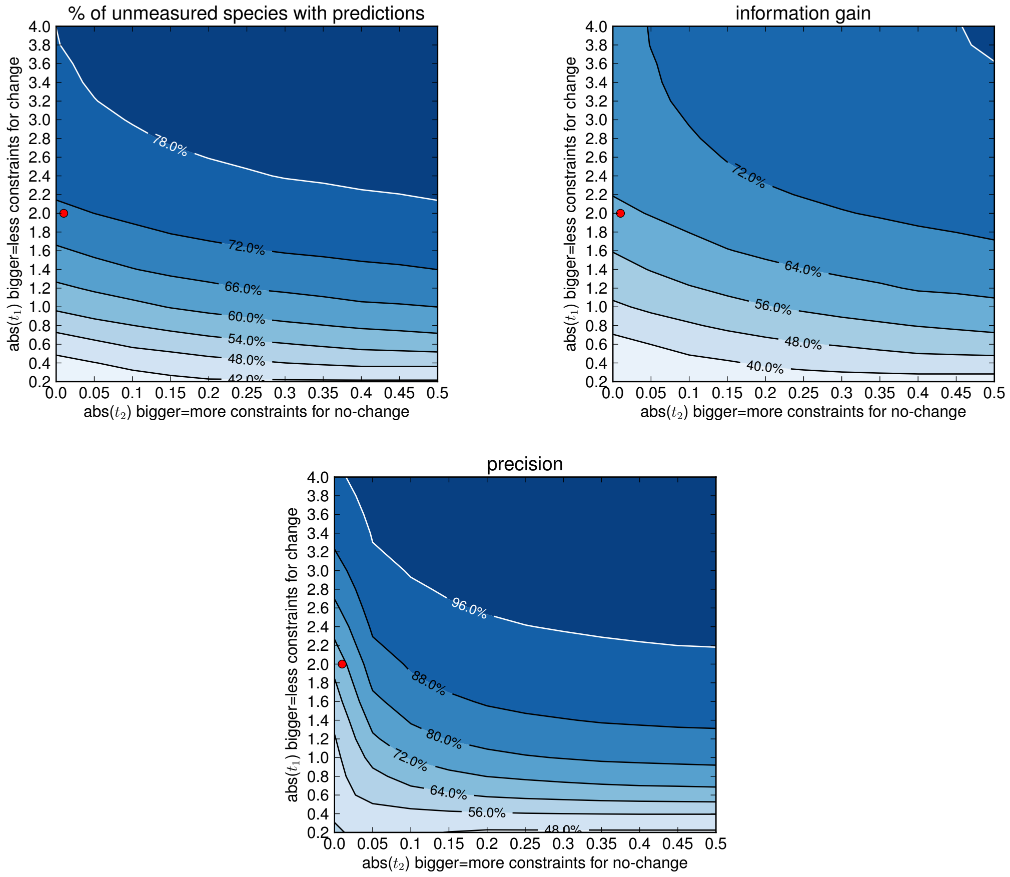

Supplementary Figure 3: Dependency of recovery rate (% of unmeasured species with prediction), information gain, and precision of predictions on the chosen thresholds. The absolute values for t_1 and t_4 as well as for t_2 and t_3 are identical. The red dot marks the thresholds chosen for the analysis in the main paper.

Following a systematic investigation of the sensitivity of recovery rate, information gain and precision on the choice of the thresholds, we present the results in Supplementary Figure 3. It is shown that recovery rate, information gain and precision do not change abruptly, but rather increase steadily towards the upper right corner where they are at the maximum. This is when most of the measurement/predictions are forced to be 0. We can identify the following 3 extreme cases:

1. **All measurements discretized as 0-change.** If one chooses the absolute values of threshold t_2 and t_3 too large all measurements will be discretized as 0-change which has exactly one trivial solution, namely the fully inactive network where nothing changes (the upper right corner in the plots in Supplementary Figure 3). Paradoxically, this results also in the most information gain because the behavior of all nodes is determined. While this is a consistent behavior it is of course not very useful and normally we can assume that at least some (input) nodes have been perturbed in the experiment which also must lead to some downstream effects in the network.

2. **All measurements discretized as weak observations.** If one chooses the absolute value of thresholds t_1 and t_4 too big many measurements will be discretized as uncertain decrease (resp. uncertain increase). In this case we give the least information into the method. This information will most likely contain only very few inconsistencies which have to be resolved by the repair process. Also it contains the least constraints and therefore will result in high number of solutions and a relatively small information gain.

3. **All measurements discretized as strong observations.** If one chooses the threshold t_1 and t_2 (resp. t_3 and t_4) very close all measurements will be discretized as strong-decrease (resp. strong-increase). In this case we give the most information into the method. This information will have more inconsistencies than in the 2nd extreme, which will be resolved by the minimal repair process. Also it contains the most constraints and therefore will result in fewer number of solutions and a relatively high information gain.

Our recommendation is to choose thresholds such that one compromises between the 2nd and the 3rd case. With uncertain observations the user can control which observations constitute less constraints rather than letting this decision be made by the minimal repair procedure. In the ideal case for each measured species a variability analysis could be performed to determine the threshold for each species individually. Often the experimentalist have insight in which observations can be trusted and for which species the measurements are highly volatile.

Inconsistency index and prediction results for *E. coli* using discretization thresholds ($t_1 = -1.5$, $t_2 = -0.01$, $t_3 = 0.01$, $t_4 = 1.5$)

To demonstrate the effect of choosing different thresholds on the quality of the data, inconsistency index and sign distribution, we recreated the analysis of the main document using a different choices of discretization thresholds: $t_1 = -1.5$, $t_2 = -0.01$, $t_3 = 0.01$, $t_4 = 1.5$. In the main document we used $t_1 = -2.0$, $t_2 = -0.01$, $t_3 = 0.01$, $t_4 = 2.0$.

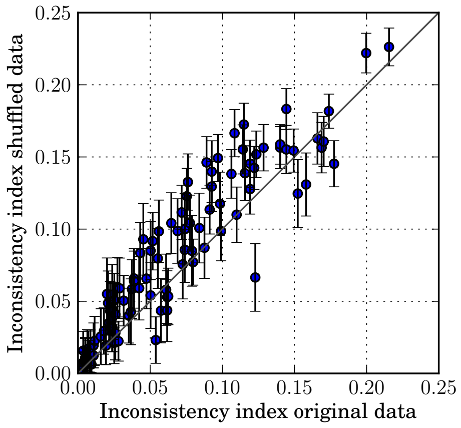

Supplementary Figure 4: Inconsistency index of the *E. coli* experiments discretized with $t_1 = -1.5$, $t_2 = -0.01$, $t_3 = 0.01$ and $t_4 = 1.5$. The x-axis shows the 105 experiments ordered after their inconsistency index getting less consistent from left to right. The y-axis quantifies the inconsistency index for the real data (black) and randomized data (red).

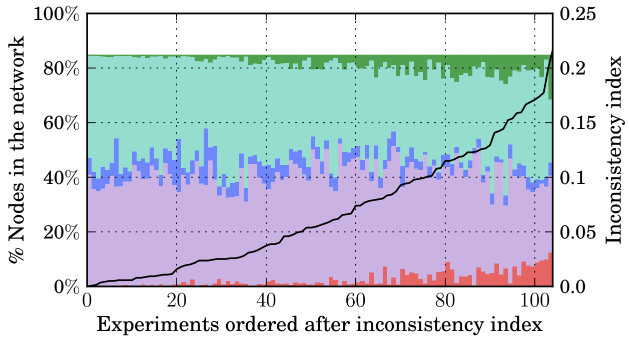

Supplementary Figure 5: Distribution of signs in the *E. coli* experimental data discretized with $t_1 = -1.5$, $t_2 = -0.01$, $t_3 = 0.01$ and $t_4 = 1.5$. The x-axis shows the 105 experiments ordered after their inconsistency index getting less consistent from left to right. The y-axis quantifies the inconsistency index for the real data (black) and randomized data (red).

In Table 2 we show the distribution of $+$, $-$, 0 and weak predictions as well as how the precision varies among the different sign consistency notions.

Prediction	+/−				0				weak predictions $\oplus/\ominus/\pm$				all predictions			
Notion	WP	FWP	SP	FSP	WP	FWP	SP	FSP	WP	FWP	SP	FSP	WP	FWP	SP	FSP
obtained using 10% of the measurements as input.																
% of unobserved nodes	0.22%	0.24%	5.34%	5.90%	0.39%	0.87%	39.50%	44.01%	10.09%	10.46%	13.38%	13.55%	10.07%	11.57%	58.21%	63.46%
information gain	0.22%	0.24%	5.34%	5.90%	0.39%	0.87%	39.50%	44.01%	3.72%	3.86%	4.94%	5.00%	4.34%	4.97%	49.77%	54.91%
precision of prediction	39.06%	37.85%	55.00%	53.91%	60.50%	68.61%	60.35%	62.05%	75.47%	74.65%	78.80%	78.71%	74.07%	73.36%	64.20%	64.78%
obtained using 50% of the measurements as input.																
% of unobserved nodes	0.70%	0.75%	6.72%	6.77%	2.44%	3.37%	61.18%	63.70%	30.92%	31.46%	7.79%	7.58%	34.07%	35.59%	75.68%	78.06%
information gain	0.70%	0.75%	6.72%	6.77%	2.44%	3.37%	61.18%	63.70%	11.41%	11.61%	2.87%	2.80%	14.56%	15.74%	70.77%	73.27%
precision of prediction	35.07%	32.67%	58.56%	60.41%	64.25%	68.82%	58.16%	58.80%	75.69%	75.61%	81.05%	81.06%	73.79%	73.79%	61.11%	61.51%
obtained using 75% of the measurements as input.																
% of unobserved nodes	0.81%	0.85%	8.07%	8.09%	3.82%	4.72%	64.54%	66.81%	40.40%	41.03%	5.78%	5.38%	45.03%	46.61%	78.39%	80.28%
information gain	0.81%	0.85%	8.07%	8.09%	3.82%	4.72%	64.54%	66.81%	14.91%	15.14%	2.13%	1.98%	19.54%	20.72%	74.75%	76.89%
precision of prediction	34.35%	31.86%	55.79%	55.79%	65.51%	67.91%	57.88%	58.34%	74.54%	74.61%	80.59%	80.60%	72.63%	72.70%	59.32%	59.64%

Supplementary Table 2: Distribution and precision of predictions under the different notions giving 10% of the *E. coli* expression measurements as input using a discretization with $t_1 = -1.5$, $t_2 = -0.01$, $t_3 = 0.01$, $t_4 = 1.5$.

5 Recovery and precision for *E. coli* cross-validation experiments

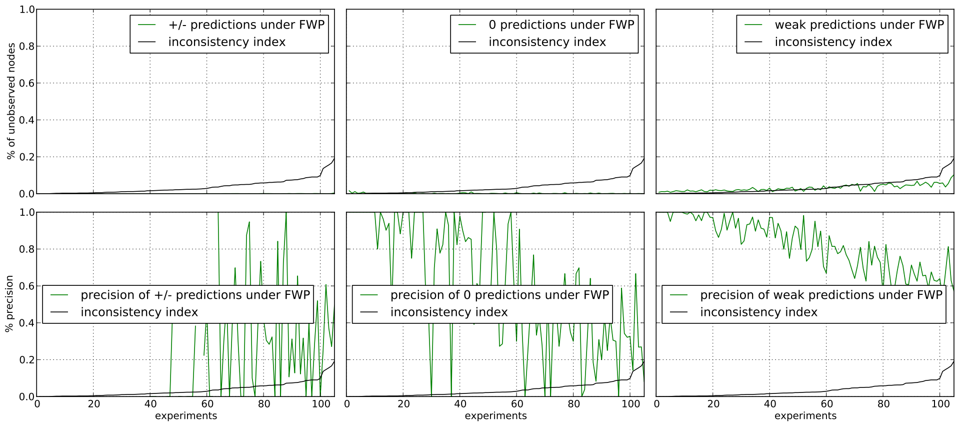

Supplementary Figure 6: % of unobserved nodes with predictions and precision for FWP given 10% of the data. The black line corresponds to the inconsistency-index of the experiments in *E. coli*.

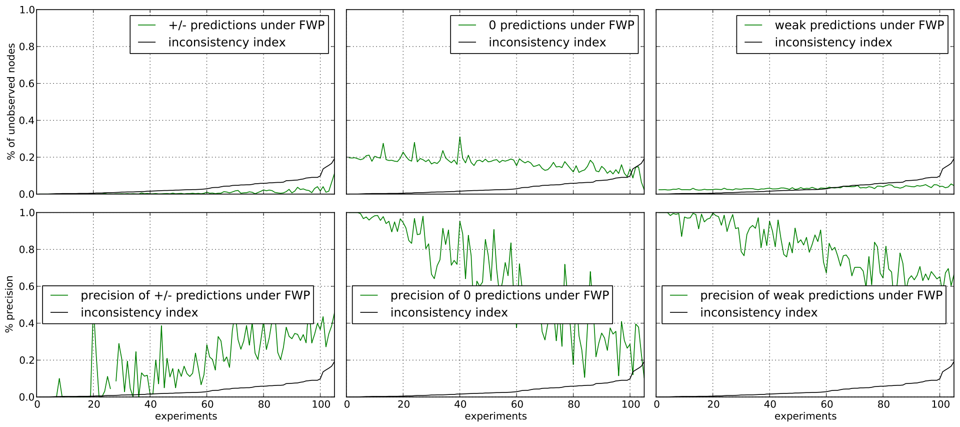

Supplementary Figure 7: % of unobserved nodes with predictions and precision for FSP given 10% of the data. The black line corresponds to the inconsistency-index of the experiments in *E. coli*.

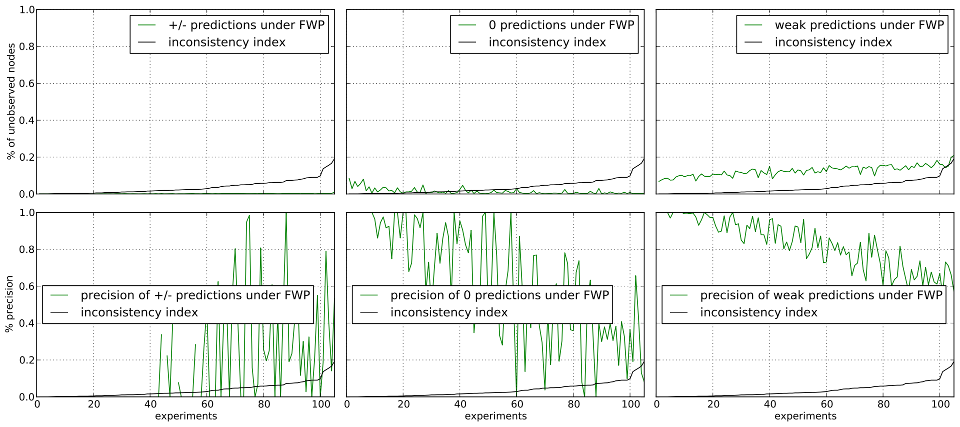

Supplementary Figure 8: % of unobserved nodes with predictions and precision for FWP given 50% of the data. The black line corresponds to the inconsistency-index of the experiments in *E. coli*.

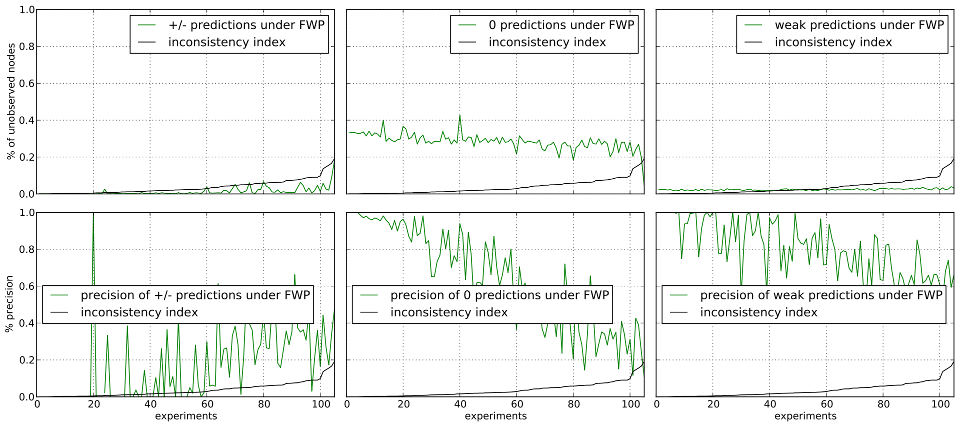

Supplementary Figure 9: % of unobserved nodes with predictions and precision for FSP given 50% of the data. The black line corresponds to the inconsistency-index of the experiments in *E. coli*.

Prediction Notion	+/− WP	+/− FWP	+/− SP	+/− FSP	0 WP	0 FWP	0 SP	0 FSP	weak predictions ⊕/⊖/± WP	weak predictions ⊕/⊖/± FWP	weak predictions ⊕/⊖/± SP	weak predictions ⊕/⊖/± FSP	all predictions WP	all predictions FWP	all predictions SP	all predictions FSP
obtained using 10% of the measurements as input.																
% of unobserved nodes	0.12%	0.13%	2.60%	2.91%	0.41%	1.19%	46.43%	53.64%	7.44%	7.74%	12.99%	13.14%	7.97%	9.06%	62.03%	69.70%
information gain	0.12%	0.13%	2.60%	2.91%	0.41%	1.19%	46.43%	53.64%	2.75%	2.86%	4.80%	4.85%	3.27%	4.17%	53.83%	61.41%
precision of prediction	31.85%	29.02%	56.28%	54.50%	76.65%	82.16%	70.69%	71.66%	82.23%	80.94%	85.19%	85.14%	80.99%	80.03%	72.97%	73.24%
obtained using 10% of the randomized measurements as input.																
% of unobserved nodes	0.14%	0.15%	2.31%	2.62%	0.35%	0.54%	47.66%	54.79%	8.66%	9.00%	12.59%	12.85%	9.14%	9.69%	62.56%	70.27%
information gain	0.14%	0.15%	2.31%	2.62%	0.35%	0.54%	47.66%	54.79%	3.20%	3.32%	4.64%	4.74%	3.68%	4.02%	54.61%	62.16%
precision of prediction	36.62%	36.94%	34.96%	34.69%	70.60%	74.27%	65.51%	67.11%	74.52%	74.56%	78.34%	78.38%	73.57%	73.74%	66.61%	67.59%
obtained using 50% of the measurements as input.																
% of unobserved nodes	0.44%	0.48%	3.06%	3.08%	2.74%	4.84%	67.93%	72.30%	28.51%	28.99%	7.27%	7.07%	31.69%	34.31%	78.26%	82.45%
information gain	0.44%	0.48%	3.06%	3.08%	2.74%	4.84%	67.93%	72.30%	10.52%	10.70%	2.68%	2.61%	13.70%	16.02%	73.67%	77.99%
precision of prediction	28.48%	24.10%	62.16%	62.14%	77.23%	82.79%	70.22%	70.97%	77.23%	82.44%	86.75%	86.80%	81.18%	81.33%	71.10%	71.57%
obtained using 50% of the randomized measurements as input.																
% of unobserved nodes	0.52%	0.58%	3.12%	3.13%	2.47%	3.42%	68.28%	72.02%	29.41%	29.93%	6.35%	6.12%	32.40%	33.92%	77.74%	81.27%
information gain	0.52%	0.58%	3.12%	3.13%	2.47%	3.42%	68.28%	72.02%	10.85%	11.04%	2.34%	2.26%	13.84%	15.04%	73.74%	77.41%
precision of prediction	32.61%	32.08%	33.96%	33.92%	74.67%	77.99%	63.85%	64.83%	78.72%	78.84%	76.43%	76.44%	77.27%	77.61%	63.15%	63.99%
obtained using 75% of the measurements as input.																
% of unobserved nodes	0.53%	0.57%	3.66%	3.67%	4.39%	7.06%	71.46%	75.37%	39.06%	39.55%	5.50%	5.12%	43.99%	47.18%	80.62%	84.16%
information gain	0.53%	0.57%	3.66%	3.67%	4.39%	7.06%	71.46%	75.37%	14.42%	14.60%	2.03%	1.89%	19.34%	22.22%	77.16%	80.93%
precision of prediction	27.26%	22.90%	58.18%	58.22%	78.66%	83.14%	70.19%	70.85%	82.14%	82.18%	85.12%	85.06%	80.81%	81.13%	70.06%	70.61%
obtained using 75% of the randomized measurements as input.																
% of unobserved nodes	0.63%	0.69%	4.12%	4.12%	4.12%	5.51%	70.68%	73.57%	39.20%	39.58%	4.76%	4.35%	43.95%	45.78%	79.56%	82.04%
information gain	0.63%	0.69%	4.12%	4.12%	4.12%	5.51%	70.68%	73.57%	14.47%	14.61%	1.76%	1.60%	19.22%	20.81%	76.56%	79.30%
precision of prediction	32.61%	31.82%	32.93%	32.89%	76.66%	79.00%	64.22%	64.87%	79.81%	79.93%	75.17%	75.21%	78.28%	78.58%	52.55%	63.13%

Supplementary Table 3: Distribution and precision of predictions under the different notions giving 10%, 50% or 75% of the *E. coli* expression measurements as input.

6 GEO/GSE codes for the experiments used

Supplementary Table 4: GEO/GSE codes for the experiments used in this paper. The category of the experimental condition is indicated with SAK, for server arrest killing, TA, for transient arrest, SG, for suboptimal growth, G, for growth, and BG, for balanced growth.

Exp. ID	Inc. Index	Description	GEO / GSE	Category
90	0	lexA060' after NOuv v s.00', MG1655	GSE9	SAK
197	0	ppk-(stationary)_ppk-(exp)	-	-
208	0	ybiQybir knockoutno Mn vs.0wtno Mn	-	-
215	0	lexA_wt	GSE9	SAK
111	0,0007	C430+0Ecb0+0IPTG v s.0C430+0Ecb0-0IPTG	-	-
14	0,0014	2min Nor(50ug/ml) beforeTreatment	GSE4367	SAK
88	0,0014	60min UVtreatmentcontrol, MG16550in Davis+0.4%glu	GSE9	TA
61	0,0021	60min after gamma treatment1', 40J, MG16550in Davis+0.4%glu	GSE4377	TA
94	0,0021	20 min @42C W3110gyrBwt v s. before upshift	GSE4417	SAK
189	0,0021	Glutamine+Gly_Glutamine+Glu	-	-
22	0,0028	50ug/ml Novobiocin 5min vs. untreated, LE234 acrD-, LB, axon5	GSE4378	SAK
112	0,0028	C430+0Ecb0+0IPTG v s.0C430+0Ecb0-0IPTG	-	-
188	0,0028	Ammonia+Gly_Ammonia+Glu	-	-
190	0,0028	Galactose_Glucose	-	-
192	0,0028	IPTG_in Gly	-	-
211	0,0028	lrp-0+0Leu v s.0lrp-00-0Leu	-	-
56	0,003	60min after UVtreatment1, 40J, MG16550in Davis+0.4%glu	GSE9	TA
78	0,003	0.1m CaCl20wash v s. before wash MG16550grown in LB	-	-
196	0,003	ppk-0(stationary)_wild-type(stationary)	-	-
45	0,0043	Amp 50min (1000ug/ml) v s.0t, M9+00.2%Glu, MG1660	GSE4357	SAK
191	0,0043	Lactose_Glucose	-	-
193	0,0057	IPTG_in Glu	-	-
241	0,0057	Balanced growth NOX+ OD 0.4 vs NOX+ OD 0.06	GSE4365	BC
214	0,007	wt+0Leu v s.0wt-0Leu	-	-
23	0,0079	200ug/ml Novobiocin 5min v s.0untreated, LE2340acrD-, LB, axon5	GSE4378	SAK
72	0,0079	stationary_1	GSE4380	TA
77	0,0079	stationary_6	GSE4380	TA
86	0,0079	8min 0.5%DMSO	-	-
134	0,0079	glu+fum_0.15_anaerob vs. glu+fum_0.4_aero	GSE4376	G
202	0,0093	tnaA2trpR2 v s.0tnaA2, W31100min +Trp 50ug/ml	-	-
127	0,0107	anrb_GluFum_stat	GSE4374	G
201	0,0107	trpR2 vs.0wt, min+.2%glucose, W3110	-	-
19	0,0114	30min Nor(50ug/ml) beforeTreatment	GSE4367	SAK
210	0,0114	ybiQybir knockoutplus0Mn vs.0wtplus0Mn	-	-
213	0,0114	lrp-0-0Leu v s.0wt-0Leu	-	-
209	0,0122	ybiQybir knockoutplus0Mn vs.0ybiQybir knockoutno Mn	-	-
199	0,0129	ppk-(4hrs n_starvation)_wild-type(4hrs n-starvation)	-	-
109	0,0136	C410+0GFp +0IPTG v s.0C410+0GFp -0IPTG	-	-
135	0,0158	glu+fum_0.30_anaerob vs. glu+fum_0.4_aerob	GSE4376	G
212	0,0158	lrp-0+0Leu v s.0wt-0Leu	-	-
200	0,0165	tnaA2trpA46PR9 v s.0tnaA2, W31100min	-	-
75	0,0172	stationary_4	GSE4380	TA
126	0,0172	anrb_GluFum6	GSE4374	G
27	0,0179	20 min @42C gyrBTsW3110gyrb234 v s. before upshift	GSE4417	SAK
203	0,0179	trpEA2trpR2 v s.0trpEA2, W31100min +Trp 50ug/ml	-	-
9	0,0186	50' trp starvation vs. 0', W3110 trpA33	GDS96	SAK
231	0,0186	Rapid Time Course of Growth Phase transition in LB Timepoint #16	-	-
84	0,0193	60min on ice in 0.1m CaCl20+015%0glycerol vs. before calcium wash	GSE4358	TA
104	0,0201	pUC19at OD 0.5 vs. no pUC19, DH5alpha	GSE4379	SG
98	0,0215	60min indol-acrylate(10ug/ml)	GSE4360	SG
114	0,0215	C410+0OGCp +0IPTG0(non-toxic) v s.0C410+0OGCp -0IPTG	-	-
185	0,0215	Glutamine+Glu_Ammonia+Gly	-	-
76	0,0222	stationary_5	GSE4380	TA
130	0,0222	glu_0.34_anaerob vs. glu_0.4 aerob	GSE4375	G
186	0,0229	Glutamine+Gly_Ammonia+Gly	-	-
107	0,0237	pUC19at OD 0.9 vs. no pUC19, DH5alpha	GSE4379	SG
71	0,0244	30min Norfloxacin(50ug/ml) gyrArparCr	GSE4369	TA
187	0,025	Glutamine+Glu_Ammonia+Glu	-	-
131	0,0265	glu_0.73_anaerob vs. glu_0.4 aerob	GSE4375	G
108	0,0287	BL21+0GFp +00IPTG v s.0C410+0GFp +0IPTG	-	-
129	0,0287	glu_0.15_anaerob vs. glu_0.4 aerob	GSE4375	G
125	0,0308	anrb_GluFum5	GSE4374	G
74	0,0323	stationary_3	GSE4380	TA
110	0,0323	BL210+0GFp +0IPTG v s. bL210+0GFp -0IPTG	-	-
132	0,0352	glu_1.02_anaerob vs. glu_0.4 aerob	GSE4375	G
73	0,0387	stationary_2	GSE4380	TA
5	0,0431	60'+50ug/ml Trp vs 0' in min med +0.2% glu	GSE4372	SAK
122	0,0431	anrb_GluFum1	GSE4374	G
142	0,0431	LB+0.2%glu 1050min OD 0.3 v s. bonner-Vogel OD 0.5, MG1655	GSE4363	G
198	0,0431	ppk-(exp)_wild-type(exp)	-	-
207	0,0431	ybiQ-ybiR-0knockout v s.0wtin LB, MG1655	-	-
128	0,0466	glu_0.08_anaerob vs. glu_0.4 aerob	GSE4375	G
124	0,0481	anrb_GluFum4	GSE4374	G
66	0,0495	30min Norfloxacin(15ug/ml) gyrArparCr	GSE4368	TA
121	0,0495	anrb_glu_stat	GSE4373	G
116	0,0502	anrb_glu1	GSE4373	G
136	0,0517	glu+fum_0.63_anaerob vs. glu+fum_0.4_aerob	GSE4376	G
6	0,0531	C410+0Ecb0+0IPTG0(toxic) v s.0C430mutant+0Ecb0+0IPTG	-	-
182	0,0538	90min recovery in LB+0.2%glu atOD 1.0from 1440min stat v s. bV0OD 0.5	GSE4364	G
161	0,0545	90min recovery in LB+0.2%glu from 1440min stat v s. bV0OD 0.5	GSE4359	G
51	0,0567	Kan 50min (1000ug/ml) v s.0t, M9+00.2%Glu, MG1665	GSE4362	SAK
137	0,058	glu+fum_0.85_anaerob vs. glu+fum_0.4_aerob	GSE4376	G
175	0,058	90min recovery in 10mm Na-p buffer (pH7.5) +00.2%glu from 1440min stat	GSE4371	G
123	0,0596	anrb_GluFum2	GSE4374	G
102	0,0610	60min indol-acrylate(15ug/ml)	GSE4361	SG
206	0,0610	ybiQ-ybiR-0knockout v s.full complementin LB, MG1655	-	-
118	0,0632	anrb_glu3	GSE4373	G
138	0,0646	glu+fum_1.07_anaerob vs. glu+fum_0.4_aerob	GSE4376	G
117	0,0725	anrb_glu2	GSE4373	G
139	0,0732	glu+fum_1.29_anaerob vs. glu+fum_0.4_aerob	GSE4376	G
113	0,0747	Overexpression OGCp in BL21+0IPTG v s.0OGCp in C410	-	-
120	0,0747	anrb_glu5	GSE4373	G
184	0,0761	M9+Glu_LB	-	-
133	0,0797	glu_1.27_anaerob vs. glu_0.4 aerob	GSE4375	G
195	0,0818	Salmonella_Ecoli2	-	-
115	0,0826	BL210+0OGCp +0IPTG v s. bL210+0OGCp -0IPTG	-	-
183	0,0833	N-C-0+Glu_0LB+Glu	-	-

Continued on next page

Supplementary Table 4 – continued from previous page

Exp. ID	Inc. Index	Description	GEO / GSE	Category
119	0,088	anrb_glu4	GSE4373	G
194	0,0905	Salmonella_Ecoli1	-	-
150	0,0941	LB+0.2%glu 1440min OD 4.7 v s. bonner-Vogel OD 0.5, MG1655	GSE4363	G
168	0,1364	90min recovery in 10mm Na-p buffer (pH7.5) from 1440min stat v s. bV0OD 0.5	GSE4370	G
32	0,1472	8min Rifampicin(500ug/ml), LB	GSE3265	SAK
39	0,1558	32' Rif5000in .5%DMSO v s.00', M9+glu, MG1655	GSE3265	SAK
204	0,1666	trpR2tnaA2,W31100minimal +Trp 50ug/ml vs.0dnaC genomiC DNA	-	-
205	0,191	tnaA2,W31100minimal +Trp 50ug/ml vs.0dnaC genomiC DNA	-	-
